# Supplementary material for: Multi-population stochastic modeling of Ebola in Sierra Leone: Investigation of spatial heterogeneity
Source: PLoS One. 2021 May 13;16(5):e0250765. doi: 10.1371/journal.pone.0250765 (PMC8118279; doi:10.1371/journal.pone.0250765)
Supplement: S1 Appendix — (PDF) [file pone.0250765.s001.pdf]

S1 Table shows the summary posterior estimates for Model 1, the most flexible model, which assumes all parameters to vary across districts. Looking at the credible intervals, the results point to a common incubation period and common infectious period. The commonality around the estimates urged us to fit a model with constant incubation period and constant infectious period, allowing only the mean transmission rate to vary. Results for this model are presented in S2 Table . The posterior estimates for this model points to equality of mean transmission rate across the districts. The estimates and DIC of these two model, presented in the results section, support the use of Model 3 for inference purpose. In fact, at least for the assumption of constant incubation period and constant infectious period, this was expected as these parameters — incubation period and infectious period — are disease specific rather than district specific.

Another important finding is that Model 1, the most flexible model, tends to overestimate the effective reproductive number, mainly in districts with less data, as can be seen in S1 Fig. The reason behind this behaviour might be due to an immense number of parameters, making the model to likely overfit the data.
