# Supplementary material for: Multi-population stochastic modeling of Ebola in Sierra Leone: Investigation of spatial heterogeneity
Source: PLoS One. 2021 May 13;16(5):e0250765. doi: 10.1371/journal.pone.0250765 (PMC8118279; doi:10.1371/journal.pone.0250765)
Supplement: S1 Table — Posterior summary statistics for Model 1 computed from a sample of 500k iterations after a burn-in of 100k. Model 1 considers that all parameter varies across districts. The model is fitted considering informative priors. 95% credible interval a reported in brackets. (PDF) [file pone.0250765.s002.pdf]

Districts	$1/\varrho_j$	$1/\gamma_j$	μ_{β_j}	$\sigma^2_{\beta_j}$
Bo	1.94 (1.67-2.2)	0.7 (0.59-0.82)	1.2 (1.13-1.22)	0.01 (0.012-0.016)
Bombali	1.35 (0.97-1.67)	0.92 (0.73-1.08)	1.25 (1.17-1.27)	0.03 (0.025-0.036)
Bonthe	1.99 (1.71-2.28)	0.88 (0.72-1.05)	1.32 (1.25-1.35)	0.01 (0.009-0.012)
Kailahun	2.38 (2.14-2.64)	0.98 (0.9-1.07)	1.27 (1.2-1.3)	0.03 (0.024-0.034)
kambia	1.96 (1.69-2.24)	0.62 (0.49-0.75)	1.24 (1.17-1.27)	0.01 (0.01-0.013)
Kenema	1.79 (1.55-2.04)	1.02 (0.89-1.18)	1.23 (1.15-1.25)	0.03 (0.022-0.032)
Koinadugu	2.95 (2.68-3.26)	0.97 (0.86-1.08)	1.25 (1.18-1.27)	0.02 (0.014-0.02)
Kono	2.28 (2.04-2.53)	0.69 (0.59-0.81)	1.22 (1.15-1.25)	0.02 (0.012-0.017)
Moyamba	1.95 (1.7-2.22)	0.71 (0.63-0.81)	1.23 (1.17-1.26)	0.01 (0.01-0.014)
Port Loko	1.77 (1.49-2.04)	0.9 (0.73-1.07)	1.19 (1.12-1.22)	0.03 (0.021-0.029)
Pujehun	2.02 (1.74-2.29)	0.91 (0.76-1.06)	1.31 (1.24-1.33)	0.01 (0.008-0.011)
Tonkolili	1.73 (1.48-1.98)	0.81 (0.7-0.93)	1.22 (1.15-1.24)	0.02 (0.017-0.024)
Western Area Rural	1.37 (1.09-1.65)	1.03 (0.87-1.19)	1.26 (1.19-1.29)	0.04 (0.03-0.043)
Western Area Urban	1.64 (1.39-1.9)	1.07 (0.92-1.24)	1.25 (1.18-1.27)	0.04 (0.028-0.038)
μ	1.83 (1.71-1.94)	0.94 (0.89-0.98)	—	—
σ^2	0.02 (0.015-0.03)	0.01 (0.009-0.014)	—	—
