# Supplementary material for: Multi-population stochastic modeling of Ebola in Sierra Leone: Investigation of spatial heterogeneity
Source: PLoS One. 2021 May 13;16(5):e0250765. doi: 10.1371/journal.pone.0250765 (PMC8118279; doi:10.1371/journal.pone.0250765)
Supplement: S2 Table — Posterior summary statistics for Model 2 computed from a sample of 500k iterations after a burn-in of 100k iterations. Model 2 assumes common incubation period and common infectious period. The model is fitted considering informative priors. 95% credible interval a reported in brackets. (PDF) [file pone.0250765.s003.pdf]

Districts	μ_β	σ^2_β
Bo	1.18 (1.11-1.24)	0.02 (0.013-0.022)
Bombali	1.26 (1.19-1.33)	0.04 (0.029-0.048)
Bonthe	1.32 (1.25-1.39)	0.01 (0.009-0.014)
Kailahun	1.28 (1.2-1.35)	0.03 (0.024-0.04)
kambia	1.20 (1.13-1.26)	0.01 (0.011-0.017)
Kenema	1.23 (1.16-1.30)	0.03 (0.023-0.038)
Koinadugu	1.25 (1.18-1.32)	0.02 (0.014-0.022)
Kono	1.18 (1.11-1.25)	0.02 (0.015-0.025)
Moyamba	1.21 (1.14-1.27)	0.01 (0.011-0.018)
Port Loko	1.20 (1.13-1.27)	0.03 (0.022-0.037)
Pujehun	1.31 (1.23-1.38)	0.01 (0.009-0.013)
Tonkolili	1.22 (1.15-1.29)	0.02 (0.018-0.03)
Western Area Rural	1.29 (1.22-1.36)	0.05 (0.035-0.058)
Western Area Urban	1.28 (1.21-1.35)	0.04 (0.03-0.048)
Incubation period	2.03 (1.94-2.13)	
Infectious period	0.93 (0.89-0.96)	
