# Supplementary material for: Multi-population stochastic modeling of Ebola in Sierra Leone: Investigation of spatial heterogeneity
Source: PLoS One. 2021 May 13;16(5):e0250765. doi: 10.1371/journal.pone.0250765 (PMC8118279; doi:10.1371/journal.pone.0250765)
Supplement: S3 Table — Posterior summary statistic for for nationwide model with constant transmission rate. The model is fitted considering the first 17 weeks of the epidemic as the assumption of constant transmission rate applies only to the early stage of the epidemic. (PDF) [file pone.0250765.s004.pdf]

| | Mean | SD | Quantiles 2.5% | 50% | 97.5% |
|---|---|---|---|---|---|
| $\beta$ | 1.27 | 0.02 | 1.22 | 1.27 | 1.31 |
| $1/\varrho$ | 1.85 | 0.07 | 1.71 | 1.85 | 1.98 |
| $1/\gamma$ | 1.05 | 0.02 | 1.01 | 1.05 | 1.10 |
| $R_0$ | 1.33 | 0.03 | 1.27 | 1.33 | 1.39 |
