# Supplementary figures and images for: Multi-population stochastic modeling of Ebola in Sierra Leone: Investigation of spatial heterogeneity
Source: PLoS One. 2021 May 13;16(5):e0250765. doi: 10.1371/journal.pone.0250765 (PMC8118279; doi:10.1371/journal.pone.0250765)

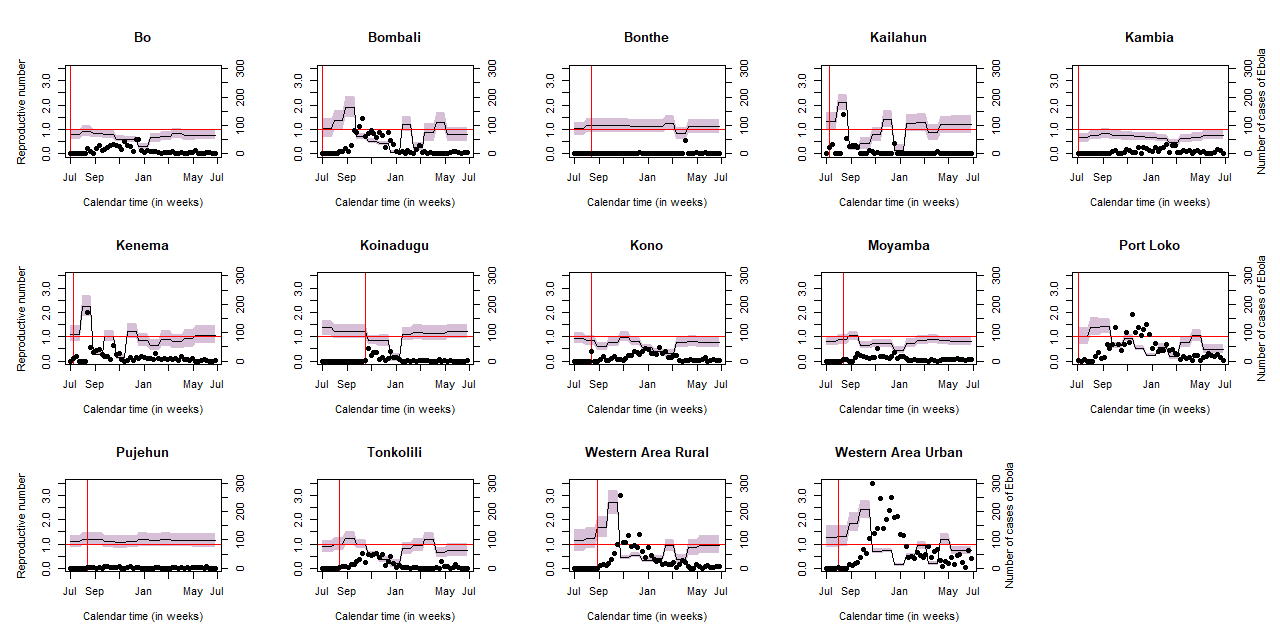

Supplement: S1 Fig — Effective reproductive number(black solid line) with its 95% credible interval. The horizontal red line corresponds to Rt = 1. The vertical red line indicates where exactly the epidemic starts. These results correspond to the model with informative priors. The curves were produced based on Model 1. This model assumes that all the model parameters vary across the district. The black dots represent the observed incidence data. (TIF) [file pone.0250765.s005.tif]

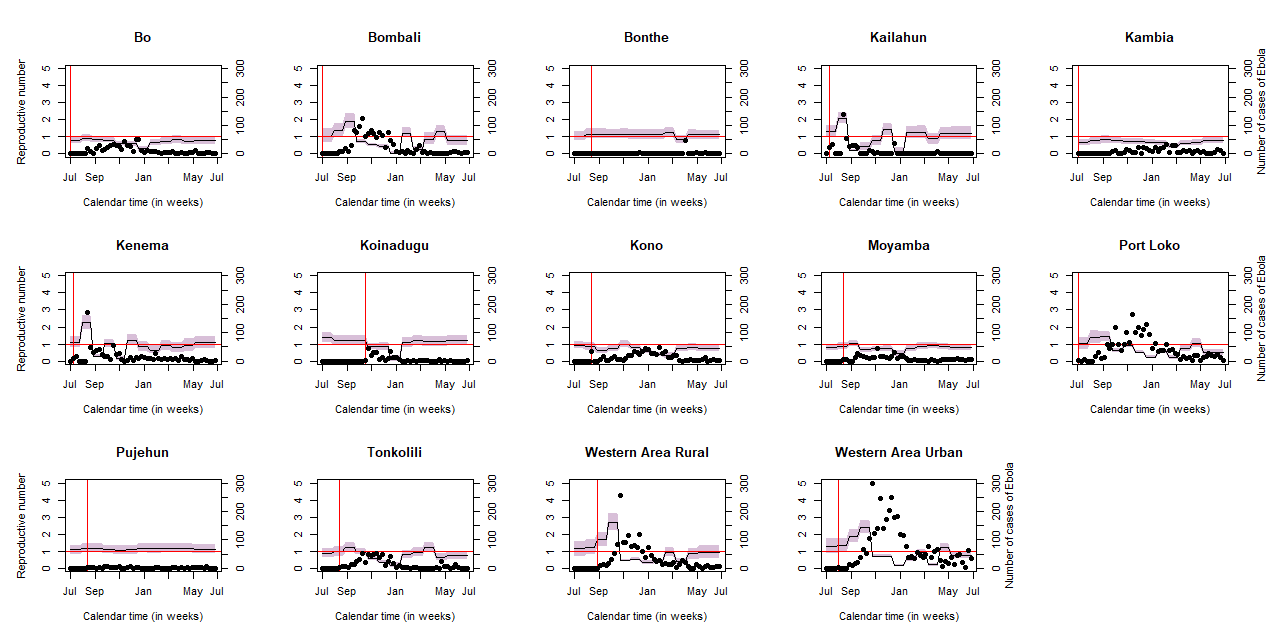

Supplement: S2 Fig — Effective reproductive number(black solid line) with its 95% credible interval. The horizontal red line corresponds to Rt = 1. The vertical red line indicates where exactly the epidemic starts. These results correspond to the model with informative priors. The curves were produced based on Model 2. This model assumes that incubation period and infectious period do not vary across the districts. The black dots represent the observed incidence data. (TIF) [file pone.0250765.s006.tif]

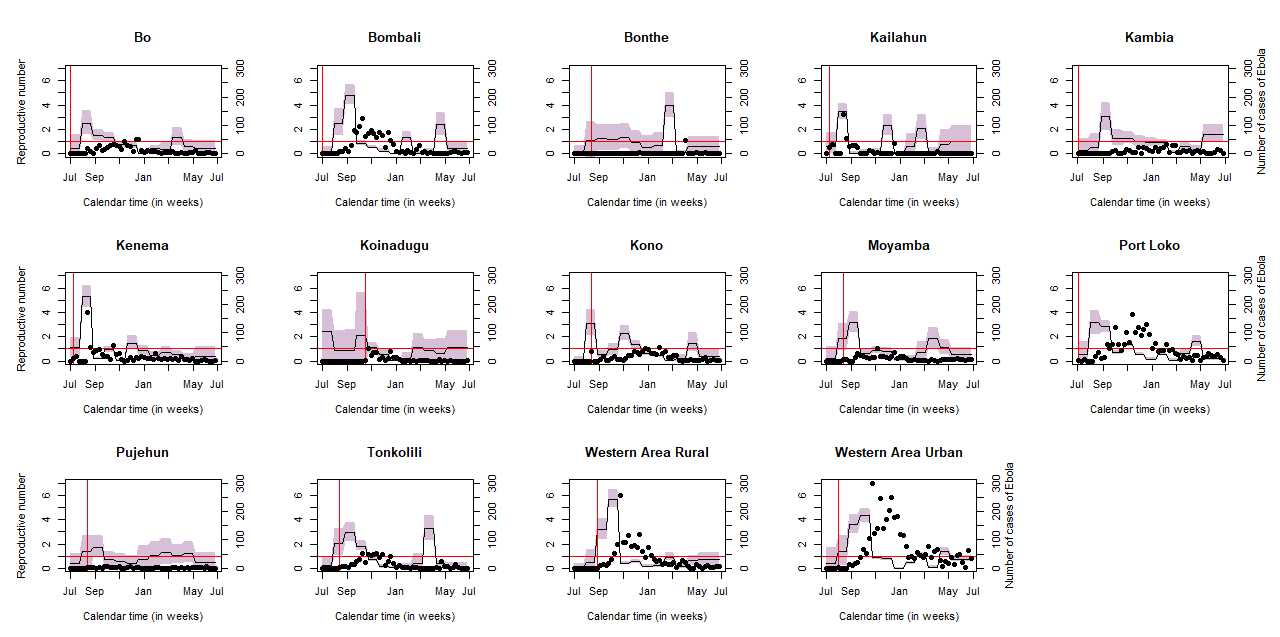

Supplement: S3 Fig — Effective reproductive number(black solid line) with its 95% credible interval. The horizontal red line corresponds to Rt = 1. The vertical red line indicates where exactly the epidemic starts. These results correspond to the model with weakly informative priors. The model assumes constant mean transmission rate, constant incubation and constant infectious period. The curves were produced based on Model 3. The black dots represent the observed incidence data. (TIF) [file pone.0250765.s007.tif]

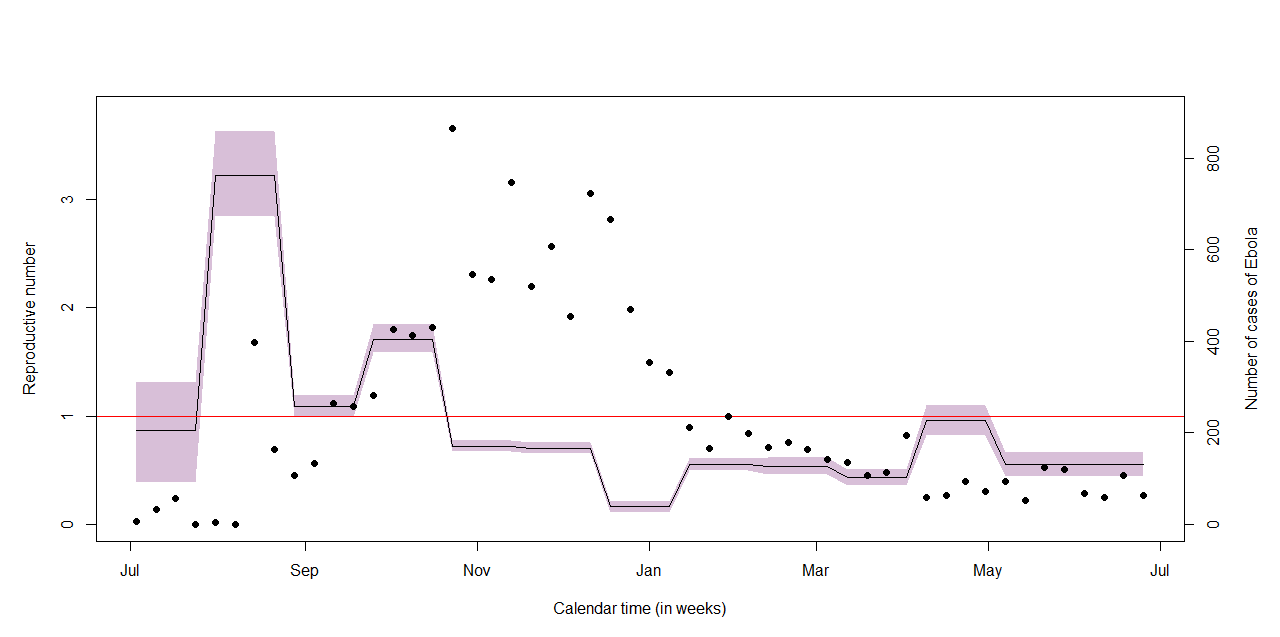

Supplement: S4 Fig — Effective reproductive number (black solid line) for the nationwide EVD data with its 95% credible interval. The horizontal red line corresponds to Rt = 1. These results correspond to the model with weakly informative priors. The black dots represent the observed incidence data. (TIF) [file pone.0250765.s008.tif]

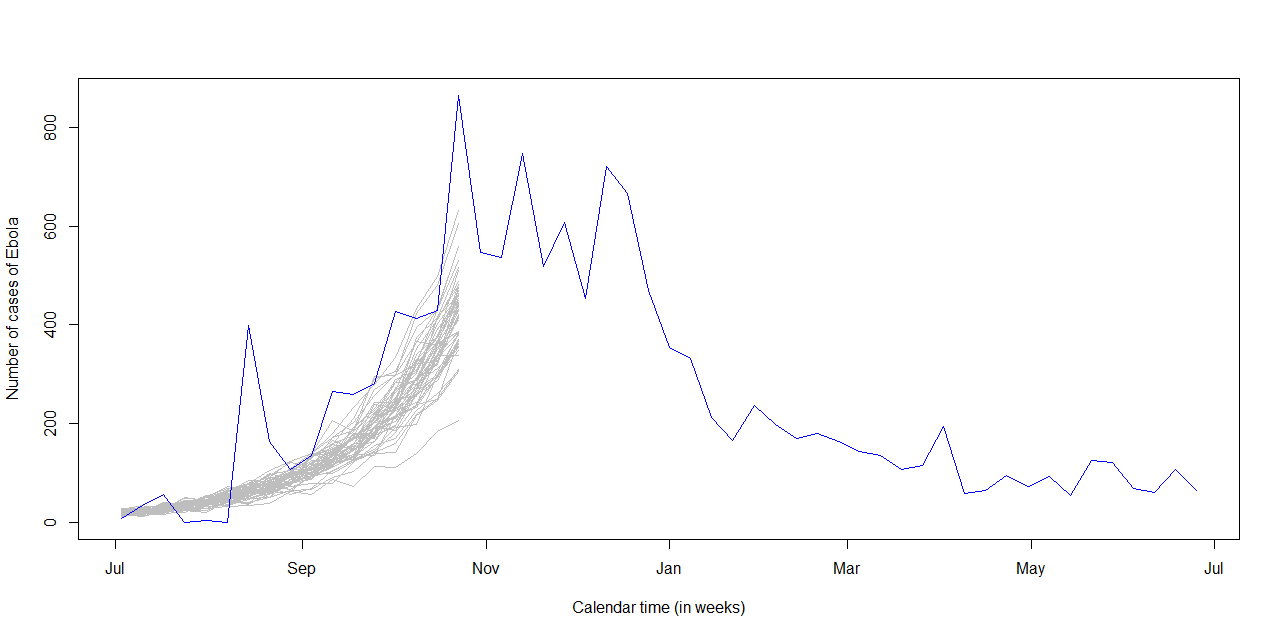

Supplement: S5 Fig — Stochastic simulation of the incidence data from the marginal posterior distribution of (β, 1/ϱ, 1/γ, E(0), I(0)). The data are simulated for the first 17 weeks of the epidemic as the assumption of constant transmission rate applies for the early phase of the epidemic. The out break reaches the peak at week 17. The blue curve represents the observed EVD incidence data. (TIF) [file pone.0250765.s009.tif]
