# Supplementary material for: Multi-population stochastic modeling of Ebola in Sierra Leone: Investigation of spatial heterogeneity
Source: PLoS One. 2021 May 13;16(5):e0250765. doi: 10.1371/journal.pone.0250765 (PMC8118279; doi:10.1371/journal.pone.0250765)
Supplement: Attachment — Submitted filename: review.pdf [file pone.0250765.s010.pdf]

Report on **Multi-population stochastic modeling of Ebola in Sierra Leone: Investigation of spatial heterogeneity**

by Rachid Muleia, Marc Aerts and Christel Faes

The manuscript analyzes data from the Ebola outbreak in Sierra Leone in 2014 by a stochastic SEIR model to infer epidemic parameters by a hierarchical Bayesian model. For this, the authors study three statistical models: in the most complex model they allow for district-dependent incubation (transition E to I) and recovery (transition I to R) times, as well as for district- and time-dependent transmission rates (transition S to E); in the second complex model they study district- and time-dependent transmission rates and set the incubation and recovery time as district-independent; in the least complex model they additionally relax the assumption of district-specific transmission rates. Using the Deviance Information Criterion, the adapted version of the Akaike Information Criterion for hierarchical Bayesian models, the authors identify the least complex model as the most suitable one to explain the data. While this result is not surprising for the disease-specific parameters, i.e., the incubation and recovery time, it is interesting to see that assuming district-dependent transition rates does not provide a better fit to the data. Potential reasons for this observation could be discussed a bit more in my opinion.

In general, I think that the 'Discussion' section is the weakest part of the manuscript. Briefly, I am missing that the authors put their results obtained from a stochastic district-specific SEIR model into context with results derived from other models and analyses. The authors do this for the effective reproductive number R_t but not for the estimated parameters. It would be interesting to see whether their detailed model provides new or other insights when compared to less detailed country-wide SEIR models. I would also suggest that they run their parameter inference on the country-wide data, i.e. accumulating all the data to a single data set, to see how the estimates compare. For more details on my concerns and suggestions related to the 'Discussion' see below.

Overall, the model and its analysis are well-explained, and the analysis is, as far as I can tell, sound. I particularly liked the 'Introduction' that nicely put this work into the context with other studies. Unfortunately, this was not the case in the 'Discussion' which is the part that needs some **major revisions** in my opinion. I have also added a small list of minor comments below that the authors might want to take into account during their revision. Once my concerns regarding the 'Discussion' are addressed, I would reconsider the manuscript for publication in *PLOS ONE.*

Major comments concerning the 'Discussion' section

1. In general, I think that the authors could do a more thorough analysis of their modeling assumptions and their implication on parameter inference. For example, how does the assumption of time dependence on the transmission rate affect their parameter estimates? To assess this, the authors could run their models with a constant transmission parameter over time.

2. Related to the comment above, I suggest to investigate how their three models perform on the accumulated data set, i.e., summing up all data sets to a country-wide data set.

3. A discussion of the parameter estimates in relation to other estimates is missing. For example, contrary to what the authors state, I think that their estimate for the incubation time is quite high when compared to other estimates that seem to be below two weeks; a fact which also motivated their prior.

4. Related to this (p9, l322): what are 'realistic' estimate? Also, I find it problematic that the more 'realistic' estimates occur in districts with fewer observations, i.e. with less data. Shouldn't it be better to have more data? This could be discussed a bit more in my opinion.

5. To address the problem of lacking information regarding the recovery (or rather death) time, I was wondering whether it would be possible to use the weekly death counts from the 2014 Ebola outbreak in Sierra Leone provided by the CDC[1]? It is not per district, but at least for the nationwide model this might be an interesting extension.

6. Lastly, it should be possible to run stochastic simulations with the obtained posterior distributions to check whether the actual data is in the tails of the distribution (which would not be good!) or somewhere centered around the mean of the simulation results (which would be desirable). This should help to assess the quality of the statistical results.

Minor comments

1. p1, lines 9-12: I suggest moving the part "Democratic Republic of Congo, formerly known as Zaire," to the first mentioning of "Zaire" (line 9)

2. p1, line 17: add "2014" after "May"

3. p2, lines 30-31: I am not sure by what is meant by "deterministic ordinary least square"?

4. *General*: There are a couple of typos throughout the manuscript, e.g. p2, l34: "estimate**s**"; p2, l45: "stud**ies**"; p2, l48: "cumulative ..." (noun missing) – I guess it should be "cases" or "counts"; p2, l67 ~~make~~; etc.

5. Fig. 1, label: I do not understand the meaning of the different starting points of the epidemic? Isn't there just one starting date? This needs some clarification here in the label (even though the authors mention it in the main text). Also, the wording for description of panel (a) is not clear.

6. p4, line 139: It seems odd to me that the authors choose e and a as the initial number of initially exposed and infectious individuals, respectively. To be consistent with the initial value of susceptibles, I suggest using e_0 and i_0.

7. p5, line 170-171: I was initially confused by the word "state" because I thought that it refers to movement of individuals between districts. To avoid this confusion, I suggest adding "epidemic" before the word or replacing it by "compartment".

8. p6, line 189: Typically, if one chooses to write out the possibilities for both genders, one goes in an alphabetical order. Therefore, it should be "her/his" instead of "his/her".

[1] `https://www.cdc.gov/vhf/ebola/history/2014-2016-outbreak/case-counts.html`

9. p6, line 190: The basic reproductive number does not only "appear" to be of importance, it "is" of importance to assess the future outcome of the epidemic. I suggest changing the wording here.

10. p7, line 223: I could trace back the prior distributions of the mean exposure time (Frasso et al. (2016), *Biostatistics*) and the mean recovery time (Lekone & Finkenstädt (2006), *Biometrics*) but not for the transmission rate. Could the authors point me to the reference and optimally state the references that motivate their hyper-prior distributions at each hyper-prior explicitly? Also, I was wondering why the authors don't take all the priors from one single paper? (In case they want to cite all the papers that they are currently citing at this point, they could state that the chosen priors are in line with estimates obtained from other Ebola outbreaks (and then cite those references).)

11. p7, line 239: Since the code is posted on github already, I suggest referring to it at this point.

12. p8, line 268: Kucharski et al. (2015), *PNAS* find a range of 0.5 (Pujehun) to 8.4 (Kailahun) and not 0.7 as stated in the manuscript.

13. Fig. 3: How are positive R_t values explained before the epidemic starts within a district? Does it have something to do with the incubation period? I suggest explaining this in the label of Fig. 3.
