# Supplementary material for: Multi-population stochastic modeling of Ebola in Sierra Leone: Investigation of spatial heterogeneity
Source: PLoS One. 2021 May 13;16(5):e0250765. doi: 10.1371/journal.pone.0250765 (PMC8118279; doi:10.1371/journal.pone.0250765)
Supplement: Attachment — Submitted filename: Response to Reviewers.pdf [file pone.0250765.s011.pdf]

Multi-population stochastic modeling of Ebola in Sierra Leone: Investigation of spatial heterogeneity

Rachid Muleia, Marc Aerts and Christel Faes

We would like to express our appreciation for the thorough review of the manuscript from the anonymous reviewers. We have addressed all the comments and suggestions from the reviewers. It considerably improved the manuscript. Below you can find point by point replies to the comments. The changes in the manuscript are highlighted in blue.

Reviewer 1

Major comments

1. In general, I think that the authors could do a more thorough analysis of their modeling assumptions and their implication on parameter inference. For example, how does the assumption of time dependence on the transmission rate affect their parameter estimates? To assess this, the authors could run their models with a constant transmission parameter over time.

   The constant transmission rate, generally, applies for the beginning of the epidemic, and for the current situation it would not be appropriate, as once the epidemic starts several interventions are implemented to stop the epidemic. Even in the early months of the epidemic, for which the assumption of constant transmission rate applies, immediate interventions measures were taken. Therefore, we did not consider a model with constant transmission rate. To support this, we also assessed, through simulation, the fit of the model with constant transmission level at aggregated nationwide level, and we observed that the simulated epidemic is not consistent with the observed data (see Figure 1). Furthermore, we also motivate why we opted for a time-varying transmission rate.We also assess the plausibility of different assumptions, district varying

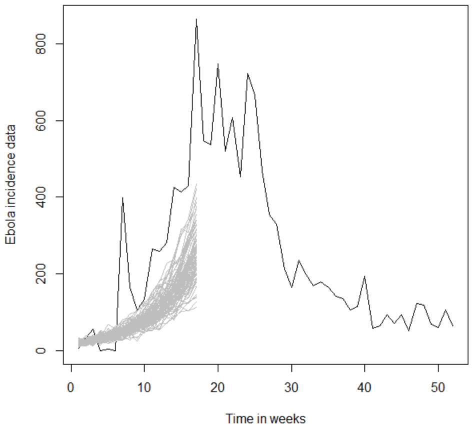

Figure 1: Simulated epidemic considering constant transmission rate.

parameters, where mean transmission rate, incubation period and infectious period are allowed to vary across the districts. Then, we consider that only mean transmission is allowed to vary across the districts. Finally, we assume that all the parameters are common across the districts. The latter appeared to most plausible given the data at hand.

2. Related to the comment above, I suggest to investigate how their three models perform on the accumulated data set, i.e., summing up all data sets to a country-wide data set.

   We have addressed this in the manuscript. Using nationwide data, we no longer have spatial dependence, hence we fitted Model 3. The model performs very well on accumulated data.

3. A discussion of the parameter estimates in relation to other estimates is missing. For example, contrary to what the authors state, I think that their estimate for the incubation time is quite high when compared to other estimates that seem to be below two weeks; a fact which also motivated their prior.

   We have addressed this in the manuscript. We noted that there was a conflict between the prior and the data. Now the posterior estimate (incubation period) is more in

line with literature. With respect to the transmission rate and infectious period, they reflect the information contained in the prior.

4. Related to this (p9, l322): what are 'realistic' estimate? Also, I find it problematic that the more 'realistic' estimates occur in districts with fewer observations, i.e. with less data. Shouldn't it be better to have more data? This could be discussed a bit more in my opinion.

   We have addressed this in the manuscript. This was actually a result of the specification of the prior distribution. Now we note that the model performs better when there is more data.

5. To address the problem of lacking information regarding the recovery (or rather death) time, I was wondering whether it would be possible to use the weekly death counts from the 2014 Ebola outbreak in Sierra Leone provided by the CDC ? It is not per district, but at least for the nationwide model this might be an interesting extension.

   In the current analysis only the incidence data are available, and to circumvent the identifibility issues resulting from lack of information on exposure and recovery (or death), we used highly informative priors. Additionally, considering only the death counts for the removed compartment would be unrealistic, as in this compartment we have those that are removed either through death or recover. If we were to consider death counts, then the model should be modified with the removed compartment divided in two (recovery and death), where we would only have available information on the deaths. The inference would still be possible, but at the cost of complexity and over-specification of the model.

6. Lastly, it should be possible to run stochastic simulations with the obtained posterior distributions to check whether the actual data is in the tails of the distribution (which would not be good!) or somewhere centered around the mean of the simulation results (which would be desirable). This should help to assess the quality of the statistical results.

We have addressed this in the manuscript.

Minor comments

1. p1, lines 9-12: I suggest moving the part ”Democratic Republic of Congo, formerly known as Zaire,” to the first mentioning of ”Zaire” (line 9)

   We have adjusted this.

2. p1, line 17: add ”2014” after ”May”

   We have adjusted this.

3. p2, lines 30-31: I am not sure by what is meant by ”deterministic ordinary least square”?

   We meant deterministic ordinary differential equations. We have corrected this in the manuscript.

4. General: There are a couple of typos throughout the manuscript, e.g. p2, l34: ”estimates”; p2, l45: ”studies”; p2, l48: ”cumulative ...” (noun missing) – I guess it should be ”cases” or ”counts”; p2, l67 make; etc.

   We have adjusted this.

5. Fig. 1, label: I do not understand the meaning of the different starting points of the epidemic? Isn't there just one starting date? This needs some clarification here in the label (even though the authors mention it in the main text). Also, the wording for description of panel (a) is not clear.

   Indeed there is one starting point. The analysis start on 3rd July 2014, the date the first case was observed in Sierra Leone (as reported by the ministery of health). In other districts, the first reported case happened at later time points, as visualized in Figure 1 in the text. We assume that there were no cases when no cases were reported. We have changed this explanation in the text (page 3).

6. p4, line 139: It seems odd to me that the authors choose e and a as the initial number of initially exposed and infectious individuals, respectively. To be consistent with the initial value of susceptibles, I suggest using e_0 and i_0.

   We have adjusted this.

7. p5, line 170-171: I was initially confused by the word "state" because I thought that it refers to movement of individuals between districts. To avoid this confusion, I suggest adding "epidemic" before the word or replacing it by "compartment".

   We have adjusted this.

8. p6, line 189: Typically, if one chooses to write out the possibilities for both genders, one goes in an alphabetical order. Therefore, it should be "her/his" instead of "his/her".

   We have adjusted this.

9. p6, line 190: The basic reproductive number does not only "appear" to be of importance, it "is" of importance to assess the future outcome of the epidemic. I suggest changing the wording here.

   We have adjusted this.

10. 10. p7, line 223: I could trace back the prior distributions of the mean exposure time (Frasso et al. (2016), Biostatistics) and the mean recovery time (Lekone & Finkenstädt (2006), Biometrics) but not for the transmission rate. Could the authors point me to the reference and optimally state the references that motivate their hyper-prior distributions at each hyper-prior explicitly? Also, I was wondering why the authors don't take all the priors from one single paper? (In case they want to cite all the papers that they are currently citing at this point, they could state that the chosen priors are in line with estimates obtained from other Ebola outbreaks (and then cite those references).)

    We have adjusted this. For the transmission rate we consider prior form (Lekone & Finkenstädt (2006), Biometrics) , and for the incubation period and infectious period

we consider information from the WHO Ebola response team, and Frasso and Lambert.

11. p7, line 239: Since the code is posted on github already, I suggest referring to it at this point.

    We have adjusted this.

12. p8, line 268: Kucharski et al. (2015), PNAS find a range of 0.5 (Pujehun) to 8.4 (Kailahun) and not 0.7 as stated in the manuscript.

    We have adjusted this.

13. Fig. 3: How are positive R_t values explained before the epidemic starts within a district? Does it have something to do with the incubation period? I suggest explaining this in the label of Fig. 3.

    In districts where the $R_t > 1$ before the epidemic starts, we observe that the credible interval contains one, indicating that the R_t is not significantly different from 1.

Reviewer 2

1. The model parameters are allowed to vary in space (in one of the model variations), but they are assumed to be independent. what is the justification? IN the earth sciences, it is common to model spatial variability using a geostatistical model, so can you explain why you chose to ignore that option?

   The motivation for considering a normal independent prior is to avoid model complexity. One could consider normal CAR priors do address spatial dependence, but in this setting it would be inefficient as we have a very limited number of regions. It is indeed the case that in earth science it's common to use geostatistical data, but that is applicable when we have georeferenced data, and in our situation the data is at district level.

2. In modeling priors, I would imagine that you will try to compile information from previous studies, but to my understanding this was not done. Please explain

   We have addressed this in the manuscript. We have included information from previous studies via the use of specific priors. The considered prior for the infectious period and transmission rate come from Lekone & Finkenstädt (2006, Biometrics), Frasso et al. (2016, Biostatistics) and the WHO Team (2014).

3. I think the authors should benchmark their study against the previous studies. Specifically, how is their model working better than previous models?

   We have addressed this in the manuscript.

4. In Line 48/49and 57 the authors state that ML cannot be used with correlated variables. This, to my understanding is inaccurate. You can take a look at publications from the earth sciences by authors such as P. Kitanidis and Y. Rubin (e.g., in Water Resources Research).

   Indeed, ML can be applied with correlated data. Example includes mixed models and other types of models. But for the case of ordinary differential equations (ODE), that are normally fitted to cumulative data, the assumption of independence is often assumed. This is not true as cumulative data are correlated.
