# Supplementary material for: Multi-population stochastic modeling of Ebola in Sierra Leone: Investigation of spatial heterogeneity
Source: PLoS One. 2021 May 13;16(5):e0250765. doi: 10.1371/journal.pone.0250765 (PMC8118279; doi:10.1371/journal.pone.0250765)
Supplement: Attachment — Submitted filename: review2.pdf [file pone.0250765.s012.pdf]

Second report on **Multi-population stochastic modeling of Ebola in Sierra Leone: Investigation of spatial heterogeneity**

by Rachid Muleia, Marc Aerts and Christel Faes

The authors have done a great job in revising the manuscript and answering to my comments. The results are much more convincing than before and put into context with previously obtained estimates. I still have a few minor comments (see attached list), but apart from these I believe that the manuscript is in a publishable state.

Remaining comments

1. Line 89 – 'for reasons that will be clear later': To me this sounds more mysterious than it should be. I suggest to state explicitly which reasons the authors refer to, e.g. modeling, statistical inference, etc.

2. Lines 118-119 and 129-131: If the transition times were not exponentially distributed, then the considered stochastic model would not correspond to the deterministic model in Eq. (1). The memoryless property is lost for non-exponential transition times and the stochastic model is not Markovian anymore so that the ordinary differential equation is not corresponding to the stochastic model. Therefore, the sentence in lines 118-119 is not entirely correct. I suggest rephrasing it to be less absolute, e.g. "We consider a stochastic discrete-time SEIR model that corresponds to the deterministic model in Eq.(1), but assumes ..."

3. Eq. (2): Why is $R(t+h)$ not defined? Is it because it is implicitly given by the constant population size assumption? But then, I think, the last line in the equation should be $S(t+h) + E(t+h) + ... = N$?

4. Eq. (5): Why not use the more common indicator function 'mathds{1}' ($\mathbb{1}$)?

5. Lines 168-170: I already commented on this in the first round of review and got confused again. I suggest either to replace 'movement' by 'transition' or 'state' by 'compartment' to avoid ambiguity with physical movement of individuals between states in a country.

6. Line 255-256: Model 1 does not result in the worst fit but in the worst tradeoff between fit and complexity. I suggest rephrasing this bit.

7. Section 'Assessing the fit': I suggest to remind the reader that Model 3 is used in this section (as is done in the following section). I first thought that the authors had used Model 1 or 2 in this section because it provides region-specific estimates. But, if I understand it correctly, Model 3 was used here as well.
